# Management of acute traumatic dental injuries in the orthodontic patient

**DOI:** 10.1038/s41415-022-4244-4

**Published:** 2022-05-27

**Authors:** Kishan Patel, Gavin Mack, Serpil Djemal

**Affiliations:** 41415119000001 Speciality Registrar in Orthodontics, King´s College Dental Institute, London, SE5 9RS, UK; 41415119000002 Consultant in Orthodontics, King´s College Dental Institute, London, SE5 9RS, UK; 41415119000003 Consultant in Restorative Dentistry, King´s College Dental Institute, London, SE5 9RS, UK

## Abstract

Knowledge of managing traumatic dental injuries (TDIs) is imperative for all dental practitioners. With the number of adults undertaking orthodontic treatment increasing, and children and adolescents alike continually being treated for orthodontics under the NHS in the UK, it is imperative that all clinicians - specialists and generalists - are aware of how to manage the orthodontic appliance in a patient presenting with a TDI in their active phase of orthodontics.

This guidance will aid practitioners in implementing pragmatic approaches to manage the orthodontic appliance in a patient presenting with a TDI. Key focus will be given on fixed appliance therapy. Case examples and flow diagrams outlining best practice are given to manage the TDI and orthodontic appliance concurrently.

## Key points

- Provides practitioners with guidance on management of orthodontic appliances in cases of traumatic dental injuries.
- Introduces clear, pragmatic flow diagrams and cases outlining the management of fixed appliances in acute traumatic dental injuries.
- Points practitioners to further relevant guidance in the management of traumatic dental injuries in the orthodontic patient.

## Introduction

The UK *Children's Dental Health Survey* in 2013 revealed that 12% of 12-year-olds and 10% of 15-year-olds had suffered a traumatic dental injury (TDI).^1^ It has been previously reported that certain types of malocclusions increase the risk of TDIs, with an increased overjet increasing the incidence by two to three times.^2^ It has therefore been suggested that one of the prime indications for early correction of a Class II malocclusion is to reduce the chance of a TDI.^3^ An increased overjet also features highly on the Index of Orthodontic Treatment Need (IOTN),^4^ which is used for prioritisation in the provision of orthodontic treatment on the NHS within the UK. The IOTN Dental Health Component ranges from 1-5 with 5 indicating the highest priority for NHS care. An overjet of greater than 9 mm confers an IOTN score of 5a, indicating a very high need for orthodontic treatment. Clinicians must therefore be mindful of both the risk of a TDI in such patients, as well as how to best manage them if a TDI is sustained during the active phase of orthodontic treatment with fixed appliances. Furthermore, with the increase in popularity of adult orthodontics in terms of both clear aligner therapy (CAT) and fixed appliances,^5^ it is also important that both general and specialist practitioners are informed of the management of patients presenting with acute TDIs within their active phase of orthodontic treatment.

This article will focus primarily on the management of TDIs during fixed appliance therapy. Consideration is also given to discussions relating to removable appliances and CAT.

## Current, established guidance

The International Association of Dental Traumatology (IADT) have been leading in the management of traumatic dental injuries for many years now and recently updated their guidelines.^6^^,^^7^^,^^8^ The most relevant updates relating to patients who sustain a TDI during active orthodontic treatment include:

- The use of 0.016 inch stainless steel wire (or fishing line) to splint repositioned teeth
- The importance of taking clinical photographs to document the injuries
- Carrying out a pulpotomy to preserve the pulp in complicated crown fracture injuries as the first line of treatment if possible
- Amoxicillin as the antibiotic of choice if this is required, although there is little evidence for the routine use of antibiotics
- Review the patients for up to five years following their TDI.

Sandler *et al.* wrote a comprehensive review regarding orthodontic management of previously traumatised teeth in 2021.^9^ Their recommendations should be considered in conjunction with these guidelines to try and provide the best care for patients.

## Orthodontic considerations in a patient presenting with acute TDIs

### Soft-tissue considerations

An impact resulting in a TDI can result in orthodontic brackets debonding and becoming embedded in the orofacial soft tissues. Therefore, in the presence of a laceration and the absence of any brackets that haven't been accounted for, a soft tissue radiograph should be taken to eliminate their presence within the soft tissues. If a bracket is seen on the radiograph (taken at 50% of the usual exposure), it should be removed under local anaesthesia.

### Root resorption considerations

Orthodontic tooth movements occur via a controlled inflammatory response involving the metabolites of arachidonic acid. Its metabolites (leukotrienes, prostaglandins and thromboxane A2) are potent mediators of inflammation. Teeth undergoing orthodontic tooth movement, therefore, are already susceptible to orthodontically-induced inflammatory root resorption.

Any additional trauma to the periodontal ligament due to an acute TDI can result in an increased risk of root resorption. In addition, a history of a previous traumatic dental injury is known to be a risk factor linked to more pronounced and clinically significant orthodontically-induced inflammatory root resorption.^10^ The timeframe on how long to wait before starting or resuming orthodontic tooth movements can be seen in Table 1.

**Table 1:** Observation periods before orthodontic tooth movement^9^

Type of traumatic dental injury	Observation period before tooth movement
Concussion	3 months
Subluxation	3 months
Lateral luxation	6-12 months
Intrusive luxation	6-12 months
Avulsion	6-12 months
Crown fracture	3 months
Crown-root fracture	3 months
Root fracture	1-2 years, or shorter if asymptomatic

## Management of TDIs in patients undergoing fixed appliance therapy

The majority of orthodontics undertaken in adolescents within the NHS involves some form of fixed appliance therapy. Therefore, consideration must be given to managing such patients with TDIs to maximise the clinical outcome of any traumatised teeth, as well as the orthodontic care.

**Fig. 1:**
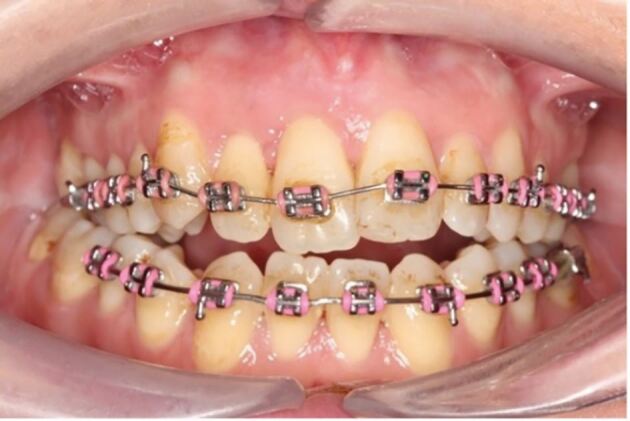
Traumatic impact causing displacement of the 11 and 12 and distortion of the archwire

Following an impact, the orthodontic archwire may become distorted (Fig. 1) or there may be loss of a bracket depending on the direction of the force and the site of impact. Any distorted archwires should be removed or sectioned to avoid unwanted orthodontic forces to the teeth which could compromise treatment outcomes and in severe cases, move teeth out of their bony envelope. The management of distorted archwires is largely dependent on the experience and confidence of the clinician, as well as the availability of materials in the clinic.

### Avulsion injuries

Due to the nature of fixed appliances acting as a splint, avulsion injuries are extremely rare, if not altogether impossible, although considerable displacements are seen. The force of the impact will be transmitted along the archwire to the adjacent teeth and is somewhat dissipated. If the impact is significant enough, theoretically it could result in the wire fracturing or all the brackets peeling off the teeth, resulting in an avulsion.

### Luxation injuries

The patient seen in Figure 1 presented one week following a TDI while in active fixed appliance therapy. The impact resulted in extrusion of the upper right central incisor (11) and upper right lateral incisor (12). The patient complained of mobility of the 11 tooth and slight tenderness to touch.

Clinically, the 11 was Grade I mobile and the incisal edge was more coronal than the 21 tooth. The 12 tooth was also slightly mobile (almost Grade I) and was not tender to percussion or palpation.

**Fig. 2:**
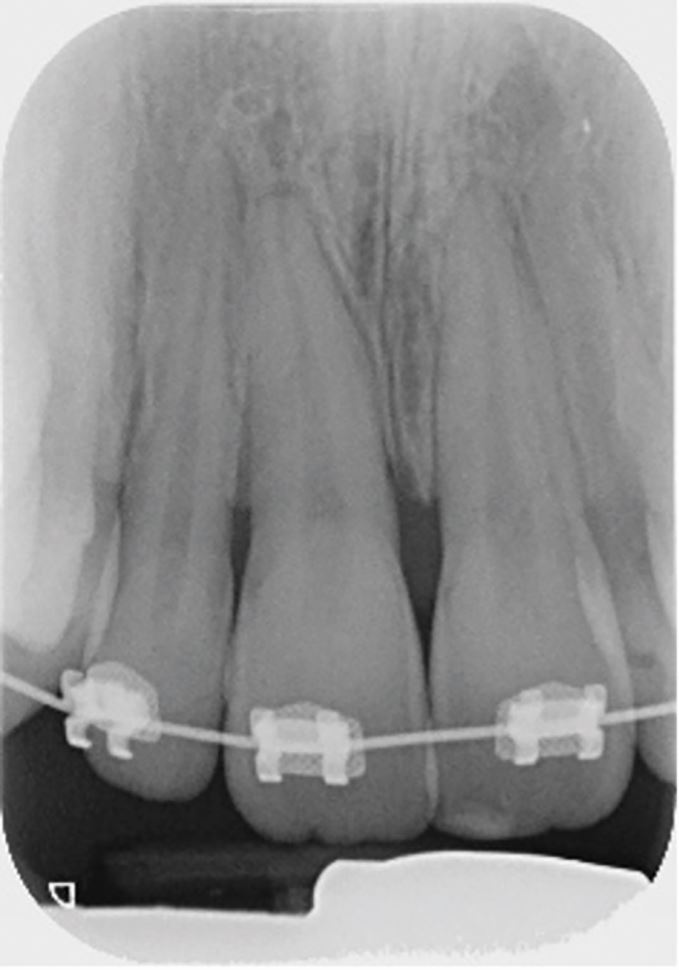
Periapical radiograph of the upper incisors showing changes in the outline of the periodontal ligament space in the 11 and 12

The periapical radiograph of the affected teeth (Fig. 2) shows a slight increase in the periodontal ligament space of the 11, mesially and apically. There is also suspicion of an increase in periodontal ligament space associated with tooth 12.

From the clinical presentation and the radiographic findings, a diagnosis of extrusion of teeth 11 and 12 was made and the decision was made to reposition the teeth digitally under local anaesthetic.

The archwire was sectioned mesial to the 14 and 21 using a diamond bur in a fast hand piece to allow inclusion of one uninjured tooth either side of the injured teeth when splinting.

**Fig. 3:**
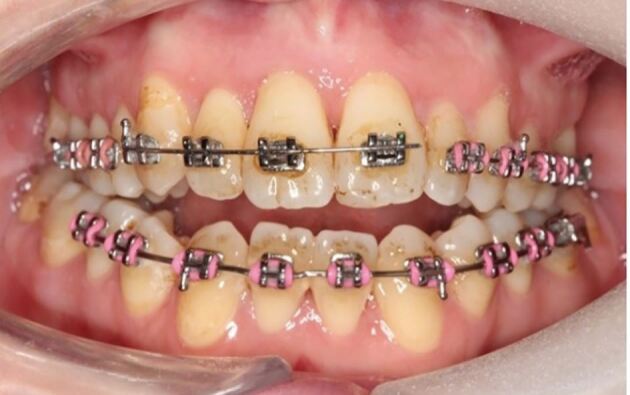
0.016 inch stainless steel wire cut and adapted to fit passively in the brackets on 13, 12, 11 and 21

A section of 0.016 inch stainless steel wire was then cut to size and adapted to fit into the brackets passively, as seen in Figure 3. Sectioning the archwire allows retention of the buccal segments while the healing process takes place around the traumatised teeth. Removal of the whole archwire is also possible but is unnecessary and the practitioner may not be confident to place a whole archwire and the risk of orthodontic relapse may increase if one is not placed.

The sectional 0.016 inch wire was then secured to the brackets with elastomeric ligatures. If the latter are not available in the clinic, a small amount of glass ionomer can be placed onto the bracket with the wire in place to encase it. This will be well retained due to the undercuts provided by the brackets, thereby holding the splint securely in place.

**Fig. 4:**
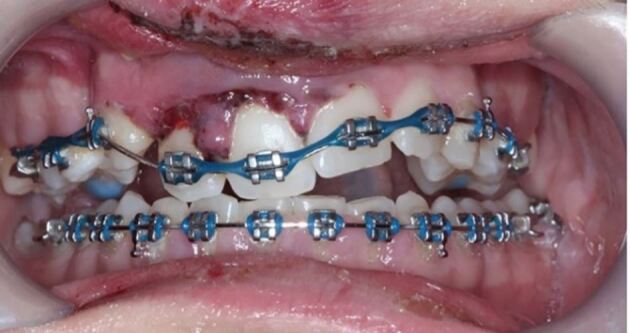
Displacement injuries to 12, 11 and 21 with severe distortion of the archwire

**Fig. 5:**
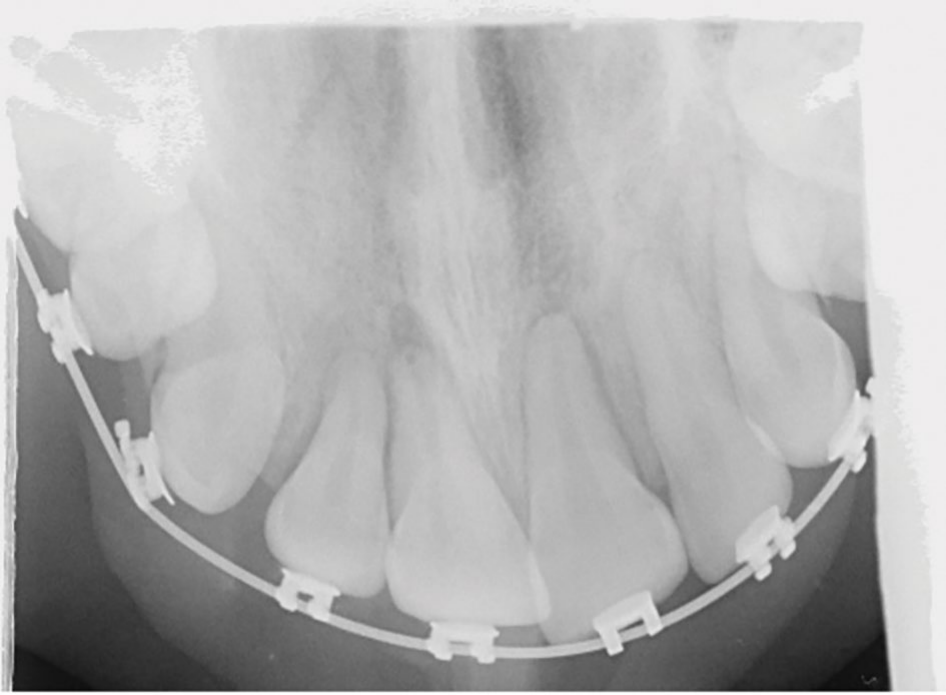
Upper occlusal radiograph showing an increase in the periodontal ligament space in the 12, 11 and 21

More severe displacement of the teeth can be seen in Figure 4, with displacement of the 12, 11 and 21. This patient presented while in upper and lower fixed appliances with rectangular stainless steel archwires. Clinically, the patient was unable to bite together into maximum intercuspation due to the palatal displacement of the upper teeth. The upper standard occlusal radiograph (Fig. 5) showed changes in the periodontal ligament space associated with the 12, 11 and 21.

**Fig. 6:**
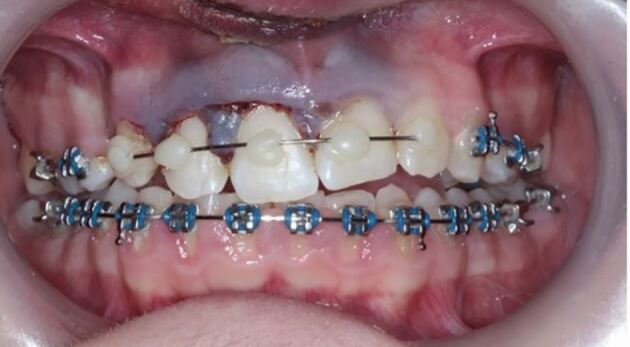
Traumatised teeth stabilised with composite and wire splint

Diagnoses of lateral luxation 12, 11 and extrusion luxation of the 21 were made and the archwire was sectioned mesial to the 14 and 23, as previously described and the brackets were carefully removed using a mosquito clip and any remaining adhesive material was polished off the teeth while stabilising the traumatised teeth using digital support. The teeth were then repositioned under local anaesthetic and were stabilised using composite and wire splint, as seen in Figure 6. Extrusion injuries are usually splinted for two weeks and lateral luxation for four weeks. If both injuries are present in the same patient, then the longest period is chosen and in line with the IADT guidelines, these teeth were flexibly splinted for four weeks. Both patients were advised to see their orthodontist six weeks after splint removal.

Reviewing patients at the appropriate time following their TDI is also important and is extensively published in the IADT guidelines for each type of injury.^6^^,^^7^^,^^8^For luxation injuries and root fractures, where tooth repositioning and splinting has been carried out, the orthodontic review can be undertaken once the splint has been removed. With crown fracture injuries, the orthodontic review can take place earlier as the review to check the sensibility of the teeth is usually after three months, when sensibility testing will be more predictable.

**Fig. 7:**
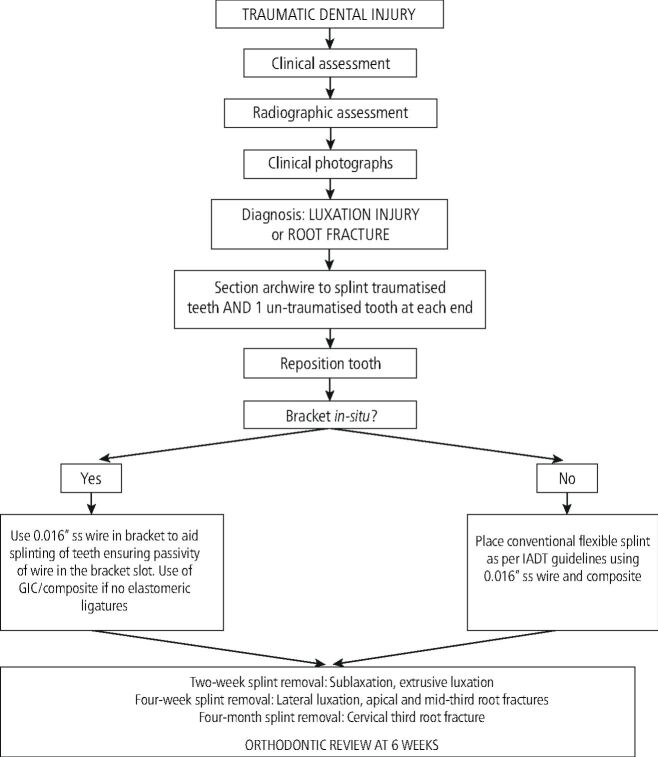
Pathway for management and stabilisation of luxations and root fractures

It would be sensible to warn patients that their orthodontic treatment will probably have to be postponed for 3-6 months and sometimes up to 12 months (in the case of severe trauma).^8^ The pathway for management and stabilisation of luxation injuries and root fractures can be seen in Figure 7.

### Fracture injuries

**Fig. 8:**
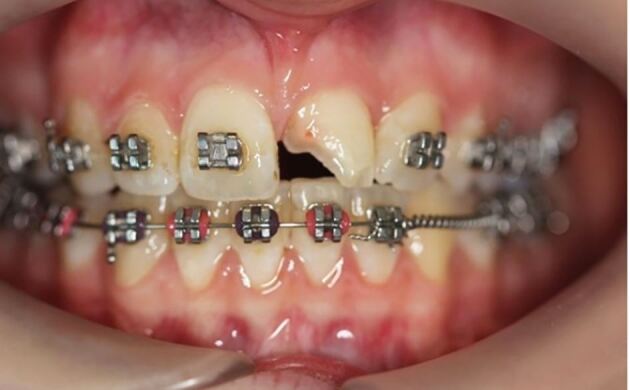
Traumatic impact to the 21 with a complicated crown fracture and loss of the orthodontic bracket

**Fig. 9:**
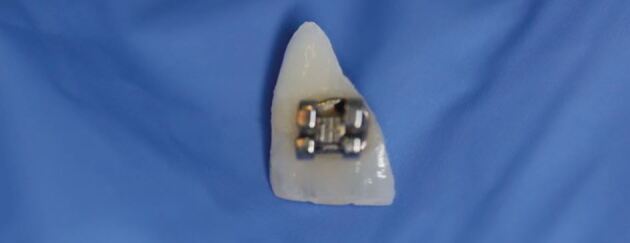
Fractured crown fragment of the 21 with the orthodontic bracket still attached

**Fig. 10:**
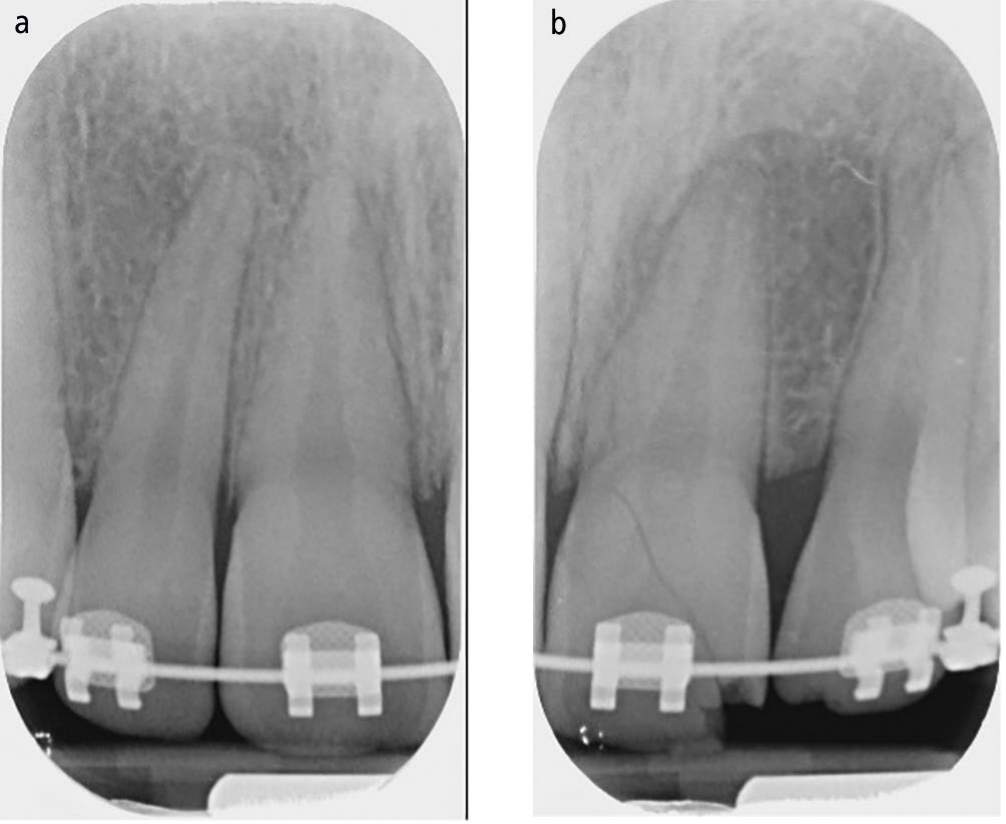
a, b) Intraoral periapical radiographs of the 11 and 12 and 21 and 22 clearly demonstrating an oblique crown fracture involving the pulp of the 21

The case seen in Figure 8 shows a complicated crown fracture associated with the upper left central incisor tooth (21) and the patient found and kept the fractured fragment of tooth safe (with the bracket still *in situ)*, which can be seen in Figure 9. The periapical radiographs (Fig. 10) revealed intact periodontal ligament spaces in the 11 and 12, clearly demonstrating a fracture line across the enamel, dentine and pulp of the 21.

**Fig. 11:**
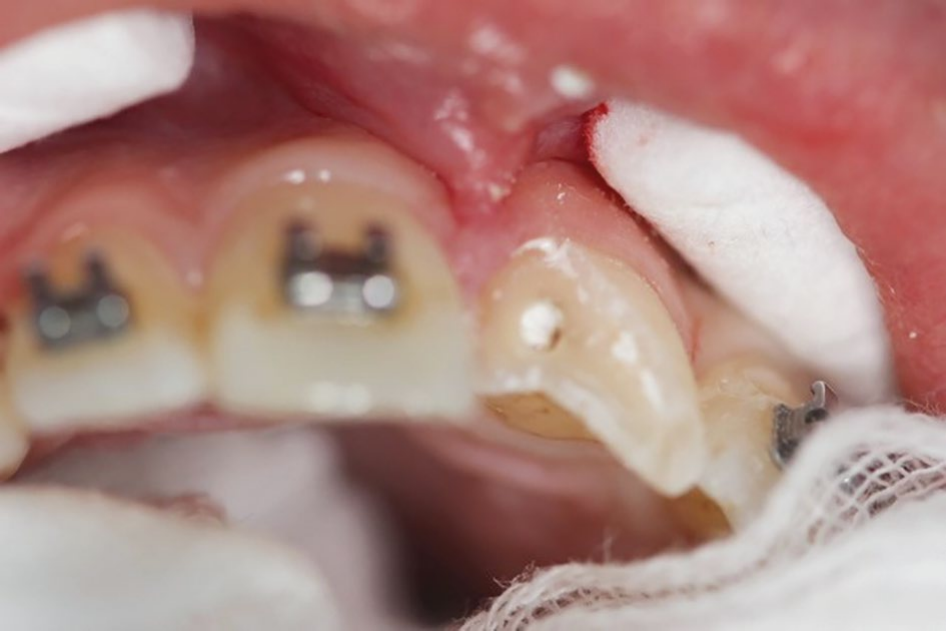
Partial pulpotomy of the 21 with non-setting calcium hydroxide

**Fig. 12:**
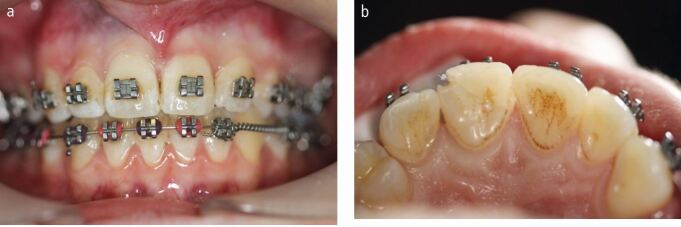
The coronal fragment of the 21 re-attached flowable composite resin. a) Labial view. b) Palatal view

A partial pulpotomy was undertaken to preserve the pulp vitality of the 21 with calcium hydroxide (Fig. 11). A glass ionomer base was placed on top and the coronal fragment re-attached with flowable composite resin (Fig. 12). The alignment of the bracket slots was used to aid correct positioning of the fractured coronal fragment.

**Fig. 13:**
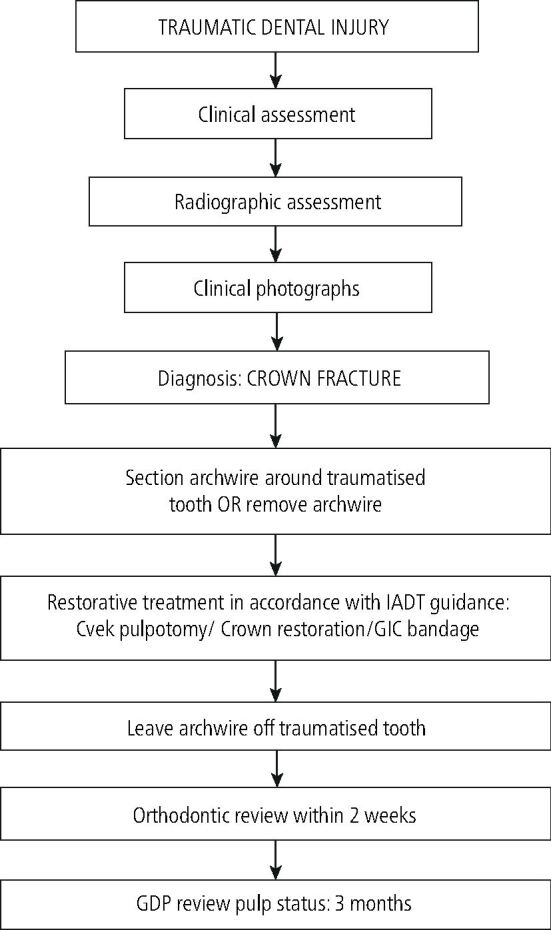
Pathway for the management of crown fracture injuries

This patient was reviewed by their orthodontist within two weeks of the TDI. The 21 was reviewed after three months following the partial pulpotomy and fragment re-attachment. Figure 13 shows a pathway for the management of crown fracture injuries.

## Removable appliances and TDIs

Patients suffering from acute TDIs with removable or functional appliances should be easier to manage. The most common functional appliance used in the UK is the Clark Twin Block. This is a removable appliance, mostly used for Class II malocclusion correction. The removable nature of these appliances means that acute management of TDIs should follow established IADT guidance. Patients should be informed not to wear their removable appliance if it is affecting their traumatised teeth until further advice from the orthodontist is sought. Any fractured wires or appliance components should be accounted for and the threshold for soft tissue radiographs should once again remain low, pending thorough clinical investigation and justification. An emphasis should be placed on making the orthodontic appliance safe.

If fixed functional appliances are *in situ*, also known as Class II correctors, these are commonly bonded to posterior teeth, which are more uncommonly associated with traumatised teeth. However, components of these appliances may become distorted, causing their improper function and in some cases, causing locking of the jaws. In such extreme circumstances, acute TDI management should be undertaken with a prompt referral to the orthodontist for management of the fixed Class II corrector.

## Clear aligner therapy and TDIs

With the increased prevalence of CAT, it is worth considering how to manage acute TDIs in these patients. Avulsion injuries are likely to be less common in light of the fact that may act as gum shields. As these appliances are removable, once again, acute TDI management should follow established IADT guidance, with active orthodontic treatment resuming depending on the type of injury. Where fractured coronal tooth fragments have been lost, clear aligners can aid their restoration by using the most recent aligner as a stent for any subsequent composite restoration. If three-dimensional scans were used to aid orthodontic treatment planning and CAT, reference to these scans can aid repositioning luxated teeth into their correct position before splinting.

## Referral details

Commonly, a patient suffering from a TDI in active orthodontic treatment will not present to their orthodontist but rather to a general dental practitioner, owing to their ready availability. Consequently, it is important that the practitioner communicates all relevant information regarding the TDI to the orthodontist in a timely manner to facilitate optimal treatment outcomes in the patient's best interests. A minimum dataset of information should be sent to the orthodontist. This can be seen in Box 1.

Box 1 Information to be sent to the orthodontic practitioner

- Baseline clinical photographs of the presenting TDI
- Baseline radiographs of the presenting TDI
- Diagnoses of TDIs (both soft and hard tissue diagnoses)
- Acute treatment undertaken to the teeth to manage the TDI
- Acute treatment undertaken to the orthodontic appliance to manage the TDI
- Any non-accidental injury concerns
- Follow-up treatment that you have arranged, including any planned endodontic procedures
- Any onward referral made to specialist care for further management of the presenting TDI.

## Conclusion

Dental practitioners and orthodontists alike should be well-versed in managing TDIs; however, managing any orthodontic appliance associated with a TDI may seem foreign to the general dental practitioner. We have developed some key algorithms and provided pragmatic approaches to aid in managing fixed appliances and other orthodontic appliances in patients within their active phase of orthodontic treatment who have sustained TDIs. This guidance should be used in conjunction with already accepted and established treatment modalities.
